# An Analysis of Broadcasting Media Using Social Media Engagement in the WNBA

**DOI:** 10.3389/fspor.2021.658293

**Published:** 2021-05-14

**Authors:** Ann Pegoraro, Heather Kennedy, Nola Agha, Nicholas Brown, David Berri

**Affiliations:** ^1^Gordon S. Lang School of Business and Economics, University of Guelph, Guelph, ON, Canada; ^2^College of Arts and Sciences, University of San Francisco, San Francisco, CA, United States; ^3^Southern Utah University, Cedar City, UT, United States

**Keywords:** social media, broadcast, new media platforms, WNBA, engagement

## Abstract

While there has been research into what teams, leagues, and athletes post on social media and the impact of post content on social media engagement, there is limited understanding and empirical research on the impact of broadcasting media on social sport consumption. There are an increasing number of new media through which sport leagues can distribute their content to fans. This research examines the impact of different broadcast platforms on game day engagement with WNBA team Twitter accounts. Using data for the 2016–2018 seasons, results indicate athlete/team quality and performance were positively associated with post engagement, underscoring the importance of the core sport product and potentially indicating that the WNBA is developing a star-driven culture similar to the NBA. In addition, broadcasting on League Pass or local TV (for home teams) and Twitter were associated with lower post engagement suggesting we have more to learn about maximizing online engagement.

## Introduction

Broadcast media has held a central role in the “nationalization” of sports or the transition of sports from a pastime to a commercial enterprise (McChesney, 1989). Historically, due to their reliance on ticket sale revenue, professional sport entities, such as baseball owners, were reluctant to embrace broadcasting of games in fear that providing free game coverage would reduce game attendance. However, these fears were misguided; rather, broadcasting revenue proved to be the greatest revenue source for professional sport organizations, with today's media deals earning professional sport leagues hundreds of millions and even billions of dollars (Pedersen, 2017). The importance of broadcast media in the sport industry should not be understated; in modern sport, “attracting spectators and media sponsorships becomes more important than the playing process because sport is now driven by profit and the market” (Frey and Eitzen, 1991, p. 508). Effectively, broadcasting has fundamentally altered and shaped the sport industry, such as determining how North American professional sports are organized, produced, and consumed (Pedersen, 2017). However, increases in cord cutting behaviors as well as alternative media distribution channels have altered the broadcast industry while providing consumers and sport leagues with access to more media options than ever before.

Recently, traditional linear television broadcasting has been challenged by the rise of *cord cutting* or the trend of canceling cable television subscriptions or landline phone connections in favor of Internet-based or wireless services. For example, in 2018, in the USA, pay television providers lost 2.9 million subscribers (Watson, 2019). Globally, this trend is remarkably consistent, with the cord cutting phenomenon reported in Asia and Europe as well (Nissen, 2016). Overall, there is a shift in viewership recently away from traditional platforms onto digital ones, a transition that is being driven “by standalone streaming services, linear over-the-top (OTT) providers, and companies like Amazon, Facebook, and Twitter, which are bidding for sports streaming rights” (Verna, 2019, n.p.). The shift to digital streaming can come in many forms: traditional television channels streaming their content directly to fans (e.g., ESPN through Disney+), owned OTT services (e.g., The Olympic Channel), league-owned apps (e.g., NFL Game Pass), dedicated streaming companies (e.g., DAZN), and social media platforms (e.g., MLB games broadcast on Facebook). Overall, this trend is challenging existing broadcasting norms, while providing professional sport leagues and organizations with more platforms than ever through which to monetize and distribute their content.

Many professional leagues, particularly non-traditional leagues, are leveraging non-linear and OTT services to effectively distribute their content and expand their audience. One league taking advantage of the increase in broadcasting options is the WNBA. Currently, the WNBA broadcasts games on ESPN2, ESPN3, NBATV, local TV, WNBA League Pass, and Twitter. Twitter has been a broadcast partner of the WNBA since the 2017 season when the league struck a 3-year deal with Twitter to broadcast 20 league games per season (Casey, 2017). The partnership between Twitter and the WNBA was renewed in 2020 with Twitter broadcasting 10 season games (Voepel, 2020). Despite the acceptance of OTT and non-traditional broadcasting channels by various sport leagues, it is currently unclear how these different broadcasting platforms impact sport consumer behavior. For instance, social media platforms were not originally designed to stream content, which may result in consumers being skeptical about embracing them as a sport media distribution channel, or, since sport consumers are motivated to use social media, such as Facebook and Twitter, for interactivity, information gathering, entertainment, fandom, and camaraderie (Filo et al., 2015), they may be an ideal fit for streaming as consumers already feel they are a source of entertainment. Alternatively, social media may not be an appropriate streaming option as consumers would rather use social media to engage and interact with content, rather than stream long-form content such as live games. Consequently, it is important to understand how different broadcasting options impact sport consumer behavior, such as social media engagement.

This research conducts an evaluation of broadcasting options by comparing social media engagement relative to the WNBA's multiple broadcast platforms. Since sport viewing has evolved into a two-way, interactive experience, engagement on social media represents an important metric to consider when evaluating broadcasting media. Online engagement is important to analyze the impacts of online and offline behavioral intentions toward a sport team (Santos et al., 2019) and brand value (Calder et al., 2018). Moreover, not only can online engagement serve as an indication of viewership but it can also spur additional viewership as non-viewers opt to tune in based on the online interactions and conversations they observe (Min et al., 2015). In addition, online engagement can further the viewing experience, such as by providing geographically diverse fans with interaction opportunities otherwise unavailable. Thus, it is important for sport leagues to understand how different broadcasting platforms correlate with online engagement, so they can make informed decisions about broadcasting medium in an increasingly saturated market. Therefore, this research analyzes the impacts of different broadcast media on game day social media engagement with WNBA team accounts during the 2016–2018 seasons.

## Literature Review

### Sport Broadcasting

Broadcasting holds an important role in the sport industry, in a large degree shaping the structure and organization of professional sport leagues in the USA. The existing broadcasting model results in broadcasting networks bidding on the rights to distribute content, such as sport programming (Evens et al., 2013). Thus, it is now commonplace for North American professional sport leagues' television rights to result in multimillion and even billion-dollar contracts with broadcasters. Since 1970, the NFL has seen a 10,000% increase in their television rights fees, while the NBA doubled their revenue for television rights since 1994 (Pedersen, 2017). Consequently, broadcasting deals are important for sport leagues as a revenue source as well as a way to distribute their content to a wide audience. However, the era of digital broadcasting has significantly disrupted the industry (Turner, 2007).

Advancements in technology have altered the relationship between sport organizations and broadcasters (Evens and Lefever, 2011), such as by increasing the number of broadcasting platforms available to sport leagues (Turner, 2007). For instance, there are a number of new distribution options available including traditional television channels streaming their content directly to fans through owned OTT services, league owned apps, dedicated streaming companies, and social media platforms. While disrupting the sport broadcasting industry, the introduction of new media options has increased the complexity of the sport media market and evolved sport teams and leagues into media entities (Evens and Lefever, 2011). There are two important implications of having more distribution options: (i) more choice for consumers and leagues and (ii) shifting sport viewing to a two-way, interactive experience.

First, emergent technologies, such as digital streaming, provide more choice to consumers and leagues (Turner, 2007); consumers have more content and more choice in how they access their content, while sport leagues have more options and control in how they distribute their content and can reach a wider audience (Fortunato, 2018). Consumers can now access content that would have been previously unavailable to them, such as subscribing to an MLB streaming bundle to access games, rather than being restricted by the broadcasting schedule of their local sports network. Additionally, as lawsuits are settled pertaining to sport media, additional purchase options are available to consumers, such as single-team options for digital media packages accessed via the Internet (Fortunato, 2018). This can result in alternative distribution channels that make content more affordable and accessible. For example, according to market research firms, the majority of cable replacement plans cost less than half of the average television bill (Willcox, 2019). Additionally, the WNBA and Twitter partnership provides free access to WNBA games for Twitter users through live streams, a partnership designed in part to increase accessibility of WNBA games (Casey, 2017). Hence, consumers have more access to content, and more choice in how they consume their content. Similarly, sport leagues have more broadcasting platforms available to them, allowing them to leverage numerous different media to reach more consumers and maximize their profits (Turner, 2007; Fortunato, 2018). This is of particular importance for smaller, less traditional leagues, such as the WNBA, which might have been traditionally limited by big broadcasters with respect to their product distribution; for example, compared with the NBA, the WNBA receives substantially less (or even no) coverage on major broadcast channels (Cooky et al., 2015). Effectively, an increase in potential broadcasting platforms provides sport leagues with more options through which to distribute their content and maximize their revenues.

Second, advancements in technology, in large part facilitated through the development of Web 2.0 technologies, have shifted the sport viewing experience from a unidirectional, static model to a two-way, interactive process (Pedersen, 2017). Sport consumers are now able to engage and interact with sport entities, including professional sport leagues, athletes, and broadcast personalities, rather than solely consume predeveloped content. This allows athletes and teams to directly communicate with their fans, rather than rely on broadcast media to facilitate their opportunities. Consequently, social media empowers athletes and allows them to develop and monetize their personal brands (Su et al., 2020), challenging the existing power structure that favored broadcasters and leagues. Similarly, new media options have forced sport teams to progress into their own media entities and provide innovative viewing experiences for fans that are more personalized and interactive (Evens and Lefever, 2011). Moreover, consumer engagement and interaction online has become an increasingly important component of the sport consumption experience as it positively influences both online and offline behavioral intentions toward a sports team (Santos et al., 2019) as well as brand value (Calder et al., 2018). Effectively, advancements in technology, particularly social media, are providing sport leagues with additional broadcasting channels as well as facilitating more interactive viewing experiences.

### Social Media

Since its introduction, social media has fundamentally altered consumer behavior and the sport industry as sport organizations, teams, leagues, and athletes have access to consumers on two-way channels, as opposed to the more traditional one-way media and advertising platforms (Achen et al., 2017). A defining characteristic of social media is the ability for users to engage with and create content. Traditional media, such as television broadcasts or newspapers, were unidirectional, with a consumer only able to read or consume content. Social media challenged this model, providing two-way, interactive communication where users could engage with (i.e., like, comment, share) and create content. It is beneficial for sport leagues to encourage fan engagement as it represents an opportunity to create a connection through unique synergies and to increase positive behaviors toward the organization (Dick and Basu, 1994; Oliver, 1999). Furthermore, professional sport organizations should increasingly focus on creating dynamic social media content and communication strategies (e.g., relevant information, improved design, and entertainment possibilities) to drive traffic and maintain a strong interactive relationship with fans (Ahn et al., 2014). In the broadcasting sphere, media providers can leverage social media to influence audience tune-in, increasing viewership numbers and advertising revenues (Min et al., 2015). Due to its role in altering consumption behavior, social media has received attention in sport management scholarship.

The diversified roles of social media in sport have been an increasingly researched topic (Filo et al., 2015), with much of this research focusing on Twitter and how individuals (e.g., fans, coaches, players, journalists, and sport media professionals) use the platform. Researchers have investigated the use of Twitter by athletes (Pegoraro, 2010), sports journalists (Sheffer and Schultz, 2010), broadcasters (Hull, 2017), and fans (Brown and Billings, 2013; Clavio and Walsh, 2014). From the sport entity's perspective, research has often acknowledged the role of social media in relationship marketing, such as discussing how it can foster relationships between consumers and sport organizations and athletes (Fisher, 2008; Abeza et al., 2013; Doyle et al., 2020). Thus, the discussion surrounding online engagement and sport entities is often focused on examining strategies, such as how different types of content are related to fans' online behaviors (e.g., liking, commenting on, or sharing a post) (e.g., Wallace et al., 2011; Anagnostopoulos et al., 2018). When considering fans, researchers have investigated areas related to explaining fan behaviors such as the motivations of sport audiences (Frederick et al., 2012) and drivers of online engagement, such as determinants of followership (e.g., team performance) (Pérez, 2013; Watanabe et al., 2015, 2016) and posting behavior (e.g., excitement levels of the game) (Lee et al., 2014). Moreover, research considering media consumption through Twitter (Lee et al., 2014) found that “Twitter works well as a complementary medium for athletes and fans—one that can enhance the experience of sport” (Kassing and Sanderson, 2010, p. 124). As such, it is important to consider social media in tandem with the broadcasting medium as it impacts the sport consumption experience.

Existing scholarship indicates that social media is often used in tandem with other media consumption including fantasy sport (Larkin and Fink, 2016; Weiner and Dwyer, 2017), Internet streaming (Collins et al., 2016), and television viewing (Gibbs et al., 2014), as well as during live games (Uhrich, 2014). Results from such scholarship indicate that social media complements or enhances the consumption experience, rather than replaces other forms of media consumption (Kassing and Sanderson, 2010). This often results in consumers using social media on a second screen, while watching their sport activity. Existing literature has found that the majority of sport consumers (77%) embrace a second screen to access social media (Cunningham and Eastin, 2017), often motivated by excitement, information, and convenience (Hwang and Lim, 2015). Moreover, as sport consumers become more excited during a game, they become more engaged on Twitter, increasing their exchange of information and opinions (Lee et al., 2014). For instance, fans bask in reflected glory (BIRG) on Twitter when their team is performing well, essentially using Twitter as an identity expression and management tool (Fan et al., 2020). Collectively, the use of social media in tandem to the viewing experience underscores the importance for sport leagues considering social media engagement relative to the broadcasting medium.

Existing scholarship has investigated social media engagement and online consumer behavior, identifying various factors that impact online behavior including post content and game characteristics. For instance, post engagement (i.e., liking, sharing, and commenting on posts) is conditional upon post content with social media users engaging with posts featuring product-related content (i.e., content containing components integral to product performance expected by the consumer such as management, head coach, star player, or team success; Keller, 1993; Gladden and Funk, 2002) more than posts containing non-product related content (Wallace et al., 2011). Similarly, for athletes, posts featuring content related to athletic performance were positively related to post engagement rates (Doyle et al., 2020). Online engagement is also tied to game characteristics such as team performance. For example, Twitter posting behavior is conditional upon game activity with fans BIRGing when their team is winning, and cutting off reflected failure (CORFing) when their team was not performing well (Fan et al., 2020). Moreover, the online behavior of followership (i.e., choosing to follow an account) on Twitter was positively impacted by team performance (Pérez, 2013; Watanabe et al., 2015, 2016). Finally, existing scholarship indicates that televising a game nationally positively impacts Twitter followership, namely the change in followers in a 24-h period (Watanabe et al., 2015). This raises the question of whether broadcasting media impact online engagement, such as national or more traditional broadcast avenues inciting engagement.

Effectively, existing scholarship often focuses on online engagement in isolation, such as the content strategies sport organizations can use to increase engagement with their posts (e.g., Wallace et al., 2011; Anagnostopoulos et al., 2018) or what factors drive consumers to engage online (e.g., follow a team's Twitter account) (e.g., Pérez, 2013; Watanabe et al., 2015, 2016). In doing so, it omits the reality that online engagement is part of an ongoing fan experience ripe with other elements, such as a second-screen viewing while watching a game on television, and its potential to be used as a metric to evaluate viewing experiences. Consequently, this scholarship uses online engagement (i.e., the number of likes, comments, or shares a post by an official WNBA account receives) as a way to evaluate different broadcasting platforms. It is an important metric to consider as online engagement impacts online and offline behavioral intentions toward a sport team (Santos et al., 2019), brand value (Calder et al., 2018), and viewership and audience tune-in (Min et al., 2015). Moreover, it is appropriate to consider as social media platforms, particularly Twitter, are complementary media that enhances the sport experience (Kassing and Sanderson, 2010).

Overall, advancements in technology and consumer preferences for cord cutting and OTT media consumption have resulted in an increasing number of media channels. This includes new features on existing social media platforms that were originally designed to facilitate the development and distribution of user generated content. Despite the importance of understanding it, it is unclear how different broadcast media will impact online engagement. This is of the utmost importance as sport leagues seek to negotiate new media rights deals in an increasingly saturated broadcasting market. Therefore, this research seeks to analyze the impact of the broadcasting medium on game day social media engagements with official WNBA team accounts. In turn, answering the following research question:

*How does social media engagement differ across various broadcast media?*

## Method

### Sample and Data Collection

Since the WNBA has taken advantage of the shifts in consumer media behavior by embracing numerous broadcast media, it is an appropriate research context. For example, the WNBA has embraced Twitter, with a correlation between team profitability and frequency of tweeting leading to the suggestion that teams should embrace Twitter and use it to update followers in-game (Shreffler et al., 2015). Moreover, the WNBA has been effective in their use of social media, such as the 2020 draft having 1.3 million minutes watched on WNBA and NBA social media accounts (Goldman and Hedlund, 2020). Additionally, as previously mentioned, the WNBA leverages numerous different broadcasting options including Twitter. Collectively, this makes the WNBA an appropriate context to leverage in the examination of broadcasting platforms. However, despite the WNBA effectively leveraging various platforms to reach their consumers, mainstream media has been less inclusive of WNBA content. For example, a review of NBA and WNBA coverage revealed that the WNBA does not receive lavish coverage while in-season, nor any off-season coverage (Cooky et al., 2015). Consequently, it is important to note that while the WNBA serves as an appropriate context for this study due to its effective embracement of Twitter and varied broadcasting platforms, the results are likely context specific, or at least most generalizable to non-traditional sports or women's sports that receive less media coverage and conversely rely on more “non-traditional” broadcasting models.

The 2016–2018 WNBA regular seasons were considered, with games serving as the unit of analysis (102 games total; 34 per season). CrowdTangle was used to obtain the interactions, defined as the number of Likes, Comments, and Retweets, of each Twitter post made by each WNBA team account on each game day. This is consistent with prior scholarship that often uses metrics such as likes, comments, and shares to capture users' engagements (Wallace et al., 2011; Cvijikj and Michahelles, 2013; Doyle et al., 2020). The 2016 season was selected as the first season as it was 1 year prior to the introduction of Twitter broadcasts and as such could provide a baseline for online engagement. The 2018 season was selected as the final season under consideration because it (i) included two seasons of Twitter broadcasts to allow consumers to adjust and embrace the broadcast medium and (ii) CrowdTangle ceased reporting on Twitter data after 2018. Since post frequency varied across teams, interactions per Twitter post were computed to compare all teams equally. Specifically, three dependent variables were computed for each game: interactions per home team post, interactions per away team post, and for both teams in each game, the average game interactions per post.

Twitter was selected as the focal, and sole platform, for three primary reasons. First, the popularity and adoption of Twitter among the sport industry and fans makes it an appropriate platform. At the time of the study, unlike other platforms, all the WNBA teams had and fully embraced Twitter accounts, allowing for engagement to be measured for all teams. Moreover, Twitter is incredibly popular among sport fans and within sport management scholarship. Specifically, Twitter is among the most popular platforms used by fans to access sport media (Billings et al., 2017) and as such as received the majority of attention in sport management social media scholarship (Abeza et al., 2015). Second, compared with other social media platforms, Twitter has a unique role of providing information and live game updates; for example, while Facebook has been likened to a team's website, Twitter is used more frequently to share news with fans (Gibbs et al., 2014). Moreover, prior scholarship has found that WNBA teams should use Twitter to provide in-game updates as well as foster relationships with their fan-base (Shreffler et al., 2015). As such, its role in game-day consumption makes Twitter an appropriate platform to use as an insight into online engagement on game days. Finally, throughout the 2016–2018 seasons, Twitter was a first mover with respect to entering the live sport broadcasting space. As such, compared with other social media platforms, Twitter is unique in its role as both a broadcaster and a social media platform throughout the WNBA 2016–2018 seasons. As such, Twitter was selected as the focal social media platform for this research.

Since this study sought to examine the impact of the broadcast medium on social media interactions, the medium(s) of each game were coded. Using the WNBA website, the medium for each game was coded as ESPN2, ESPN3, NBATV, Local TV, WNBA League Pass, or Twitter, with the potential for a game to receive multiple codes if it was broadcasted across multiple media. Since social media behavior during a game is in part a function of team popularity and quality, various control variables were considered including win percentage, championship team, and number of MVPs and all-star players on the team. Basketball reference was used to capture the win percentage of home and away teams and the count of the number of MVPs and all-star players on the home and away teams. Furthermore, market characteristics could impact the amount of social media engagements. Thus, the county populations as per the Census Bureau were included and indicator variables were added to capture if the home team had moved to a different arena that season or was in a new market (i.e., Dallas Wings in 2016 and Las Vegas Aces in 2018).

### Model and Analysis

To determine the effect of the broadcast medium on social media engagement, an ordinary least squares regression with year-fixed effects was used to estimate the panel data. This is the typical statistical method for estimating the effect of various independent variables on a single dependent variable (Watanabe et al., 2015). Specifically, we estimated *y*_*ijt*_ = β1*X*_*ijt*_ + υ_*t*_ + ε_*ijt*_ where *y*_*ij*_ was the number of interactions per post for team *i* in game *j* (tested for three dependent variables: average game interactions per post, interactions per home team post, and interactions per away team post), *X*_*ijt*_ includes measures of team quality, market characteristics, and broadcast platform variables as described above, υ_*i*_ are year fixed effects, and ε_*ijt*_ is a standard error term with a mean of zero. The results for testing the three models each with their separate dependent variable are presented below.

## Results

The descriptive statistics in Table 1 indicate that WNBA game day Twitter posts received a wide range of social media interactions from 4.72 to over 400 per post, although the majority of posts received < 100 interactions. The majority of games were on League Pass or local television with < 7% of games broadcast on ESPN2, ESPN3, and Twitter.

**Table 1:** Descriptive statistics.

**Variable**	**Mean**	**Std. Dev**.	**Min**	**Max**
Average game interactions per post	45.02	31.06	7.09	247.04
Interactions per home team post	48.65	44.51	4.72	405.94
Interactions per away team post	41.27	37.00	2.89	331.11
Win percentage home team	0.497	0.234	0	1
Win percentage away team	0.504	0.234	0	1
Champ last season (home team)	0.08	0.27	0	1
Champ last season (away team)	0.09	0.28	0	1
All-star players (total of home and away)	1.67	1.17	0	5
All-star players home	0.83	0.87	0	3
All-star players away	0.84	0.87	0	3
MVP total (total of home and away)	0.17	0.37	0	1
MVP home team	0.08	0.27	0	1
MVP away team	0.09	0.28	0	1
First season in new market	0.06	0.23	0	1
New arena	0.22	0.41	0	1
Population county	3,069,594	2,758,606	269,033	10,200,000
ESPN2	0.07	0.25	0	1
ESPN3	0.07	0.25	0	1
NBATV	0.24	0.43	0	1
Twitter	0.06	0.24	0	1
League Pass/local TV	0.87	0.34	0	1

**Figure 1:**
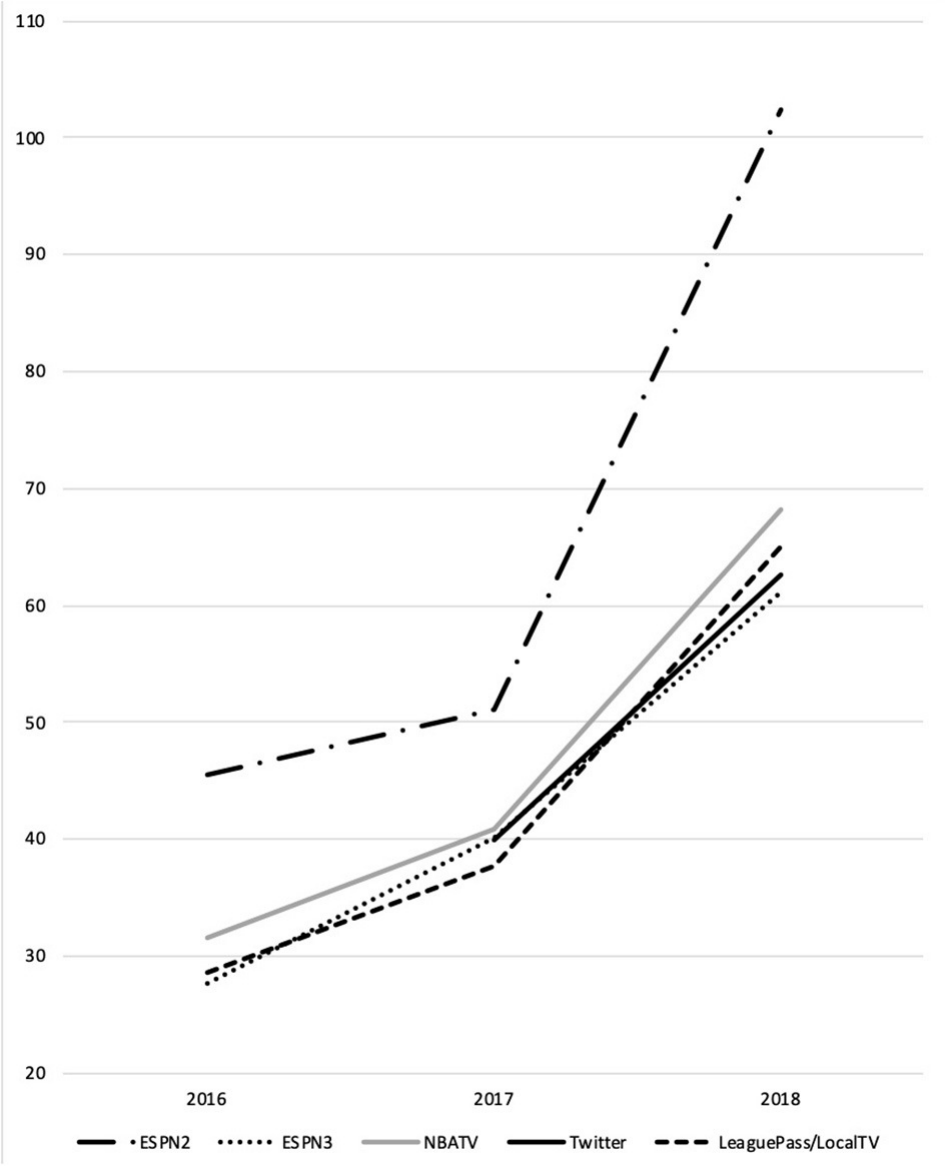
Average of home and away team interactions per Twitter post, WNBA 2016−2018.

For all three models, game day WNBA social media posts saw an increase in interactions over the 2016–2018 seasons, with the sharpest increase in interactions on ESPN2. In 2018, the ten games with the most interactions on ESPN2 featured six games with the previous season champion Minnesota Lynx and home teams with high numbers of all-star players like the Phoenix Mercury, Seattle Storm, and Los Angeles Sparks (Figure 1).

A Breusch-Pagan test for heteroskedasticity indicated the need for robust standard errors in all three models (*p* < 0.001). Variance inflation factors for all three models showed no concerns with multicollinearity (VIF < 2.0). As expected, control variables pertaining to team quality influenced social media engagements (Table 2). Specifically, being the league champion the previous season (*p* < 0.001), the number of all-star players (*p* < 0.05), and the presence of an MVP (*p* < 0.001) were all associated with higher interactions for home team posts, away team posts, and the average of both (i.e., in all three models). Results also consistently showed across all three models that social media interactions for the 2016 and 2017 seasons were lower than the 2018 reference season (*p* < 0.001), indicating an increase in social media engagement over the sample years. A new team in a market was associated with more interactions for home games (model 2) but not when the team plays on the road (model 3).

**Table 2:** Effect of broadcast type and team quality on interactions per Twitter post.

**Variable**	**Model 1 Average game interactions per post**	**Model 2 Interactions per home team post**	**Model 3 Interactions per away team post**
Win percentage home team	−5.72	−12.70^*^	−5.13
Win percentage away team	−12.04^**^	−1.23	−17.11^**^
Champ last season (home team)	32.82^***^	58.58^***^	5.11
Champ last season (away team)	25.70^***^	4.71	52.25^***^
All-star players (total home and away)	1.98^*^		
All-star players home		6.25^***^	0.40
All-star players away		−2.06	4.26^*^
MVP total (total of home and away)	17.37^***^		
MVP home team		36.75^***^	6.39
MVP away team		−0.48	29.23^***^
First season in new market	11.04^*^	31.23^***^	−2.15
New arena	4.78	5.95	1.47
Population county	0.00	0.00	0.00
ESPN2	0.43	−2.70	−1.99
ESPN3	−0.16	−5.94	7.27
NBATV	1.00	−0.81	2.69
Twitter	−2.50	−11.47^#^	6.54
League Pass/local TV	−6.99	−17.14^**^	2.63
2016	−36.47^***^	−38.53^***^	−31.47^***^
2017	−26.68^***^	−26.44^***^	−21.97^***^
Constant	67.01^***^	76.77^***^	54.15^***^
*N*	590	590	590
*R*^2^	0.447	0.382	0.330

# *p < 0.07*;

* *p < 0.05*;

** *p < 0.01*;

*** *p < 0.001*.

A higher home team winning percentage was associated with lower interactions on the home team posts (*p* < 0.05), all else equal. Similarly, higher away team winning percentages were associated with lower interactions per away team post (*p* < 0.01), all else equal. There are a few explanations for this seemingly contradictory result. First, there is a correlation between win percentage, championships, star players, and MVPs. In this context, when dummies for championships and stars are held constant, winning percent is related to lower social media interactions, but when accounting for the full set of team quality variables, winning percent simply adjusts the overall measure of interactions to be more precise. Alternatively, the first few games of the season include teams with win percentages of zero (e.g., the team lost their first game) and with a short WNBA season, this might skew the parameter.

When looking at the broadcast medium, broadcasting the game on League Pass and local television (*p* = 0.0021) is associated with lower social media interactions, but only for home team posts (Model 2). This might be explained by a team's most ardent (and social media engaged fans) being engaged at the game or at watch parties and thus tweeting less. It could also reflect that League Pass blacks out any local game that is already being broadcast on ESPN or ABC. League Pass viewers can watch these games after the live broadcast which could result in a smaller audience that is able to watch, and thus interact, on game day. Finally, in considering the recent innovation of broadcasting WNBA games on Twitter, the results show there is a decrease in home team social media interactions for games broadcasted on Twitter (*p* = 0.0683).

## Discussion and Implications

Between 2016 and 2018, the WNBA team Twitter accounts saw an increase in post engagement indicative of a greater demand for the WNBA. Given the importance of online consumer engagement via its impact on online and offline behavioral intentions toward a sport team (Santos et al., 2019), on brand value (Calder et al., 2018), and on increasing viewership and audience tune-in (Min et al., 2015), we sought to more clearly understand what factors impact game day engagement with official team Twitter accounts, with a particular emphasis on the impact of the broadcasting medium. As per our results, there are two notable sets of drivers of Twitter engagement with official WNBA team accounts on game days: (i) team/athlete quality and performance and (ii) broadcasting medium.

First, our results indicate that several measures of team quality were significantly associated with social media post engagements, namely the presence of an MVP player, the number of all-star players on a team, and being the league champion the previous season. The positive impact of team performance on post engagements aligns with prior scholarship indicating that Twitter followership is positively impacted by team performance (Pérez, 2013; Watanabe et al., 2015, 2016). The positive relationship between engagement and team performance also aligns with existing scholarship that has found consumers becoming increasingly engaged online as they become more excited during a game (Lee et al., 2014), including using social media as an identity expression and management tool (Fan et al., 2020). Moreover, the impact of star player on post engagement is consistent with previous research on demand in social media following (Watanabe et al., 2017). Overall, fans react more to star players than winning, a result which runs counter to attendance demand (Berri et al., 2004). This suggests that social media post engagement has different antecedents than in-person attendance and that perhaps the WNBA is developing a star-driven fan culture similar to the NBA (Jane, 2016).

In doing so, this research contributes to our theoretical knowledge with respect to the value of star players and team success with respect to online engagement by considering a different context. While prior scholarship has considered various sport contexts, such as professional soccer leagues (Pérez, 2013) and the MLB (Watanabe et al., 2015, 2016, 2017), this research considered the WNBA which is unique as it is a women's league and deemed a “untraditional league.” The alignment between prior scholarship and our results suggests a commonality with respect to drivers of online engagement across sport contexts and men's and women's sport at least at the professional level. Practically, the importance of these drivers underscores the importance of sport teams and leagues prioritizing the quality of their product and their star players as these factors are tied to social media engagement. It also suggests that leagues and teams can share best practices with respect to social media strategies as there is evidence of core elements that drive engagement, such as star players and team performance, across sport teams and genders at the professional level. For instance, the importance of star players and team performance with respect to online engagement for the WNBA is consistent with that of the NBA, suggesting that the WNBA can look to the NBA for advice and best practices with respect to social media strategies.

Second, this research explores the impact of the broadcasting medium on Twitter post engagement rates. Second screens are undeniably a vital platform for fans to communicate (Cunningham and Eastin, 2017), especially as games become more exciting (Lee et al., 2014). Yet, the results suggest that despite increased second screen interaction during traditional broadcasts (Voorveld and Viswanathan, 2015; Smith et al., 2019), when a WNBA game was broadcast on League Pass or local TV, there were less engagements with home team posts. This could be because home team fans are at the game, thus tweeting less (Smith et al., 2019), at watch parties or local bars viewing with their friends, thus not needing social media for identity expression and management purposes, or because there are times when League Pass locally blacks out a game, thus reducing the number of home team fans who can engage with their local team posts.

Practically, this lends additional evidence to the notion that leagues' broadcasting rules may be punitive to fans. Often leagues embrace policies designed to maximize broadcasting revenue for their teams, at times at the fans' expense (Fortunato, 2018). This research suggests that broadcasting avenues or rules, such as broadcasting home games on League Pass, may negatively impact online engagement, an important component of the fan experience and one that has important managerial implications (e.g., impacts sport teams' brand value; Calder et al., 2018). Therefore, this research highlights the complexities of broadcasting decisions in the digital era, while suggesting that sport leagues consider online engagement in the broadcasting decision making process as it is an important element of media consumption.

We also found from our results that when a game is broadcast on Twitter, posts on WNBA team accounts receive less engagement than when the game is broadcast on other platforms (i.e., ESPN2, ESPN3, NBATV, or League Pass/local TV). This intriguing result could be explained by users adapting to new methods of broadcasting or that it is difficult for fans to multitask and navigate watching and tweeting on the same platform. Alternatively, this result could be explained by Twitter providing viewing access to individuals who otherwise would have been reliant on Twitter for game updates as they had no broadcast option available to them. Social media is frequently used as a source of information (Filo et al., 2015), with fans engaging online to exchange information and opinions (Lee et al., 2014). Consequently, fans who are unable to view a game through its broadcast channel (e.g., do not have cable or away from a TV such as commuting during a game), may have previously relied on game updates through social media. Hence, when Twitter broadcasts the game, it may provide such fans with a viewing option, eliminating their need to check Twitter for updates, in turn limiting their post engagements. Practically, this suggests that the introduction of new broadcasting media disrupts the existing broadcasting model and impacts fans' experiences, such as their online engagement on game days. The WNBA, and other sport leagues, should be cognizant that not all new media distribution options will be comparable with more “traditional” broadcasting options and that should be accounted for in their decision-making processes.

Regardless of the reason why, our results indicate lower social media engagement rates for official WNBA team game day posts when the game is broadcast on League Pass or local TV (for home teams) and on Twitter. However, this is not to mean that national broadcast media, such as NBATV, ESPN2, or ESPN3, indicate higher levels of online engagement; with these broadcast types not being significantly associated with online engagement. This suggests that there may be a “standard” engagement level on game days that is only impacted by market disruptors (i.e., Twitter providing access to games free to users who may not have otherwise been able to watch a game). This also suggests that if a WNBA team seeks to increase online engagement, it might require a significant disruption to the existing broadcasting structure (i.e., changing league schedules, prioritizing female content, etc.). Overall, since social media engagement can positively influence online and offline behavioral intentions toward a sport team (Santos et al., 2019) as well as positively influence viewership and audience tune-in (Min et al., 2015) and brand value (Calder et al., 2018), it is important for sport leagues to consider team performance factors as well as the broadcasting medium.

## Limitations and Future Directions

This research examined factors associated with official WNBA team's game day social media posts, with a particular emphasis on understanding the influence of the broadcast medium. Though valuable and informative, our insights are limited by the highly dynamic nature of social media platforms. Additional years of data would help us better understand whether post engagement continues to remain lower when the game is streamed on Twitter relative to other broadcasting options. Twitter was the sole social media platform considered in this research not only in part due to its popularity and role as an information source for games and news updates but also because it was the sole social media platform that broadcast live games through the 2016–2018 WNBA season. Though Twitter was an appropriate platform to focus on, results could be limited by only measuring online engagement on one platform. Since platforms are unique and serve different purposes for consumers (e.g., Facebook as a team website and Twitter as a timely, information source for game updates; Gibbs et al., 2014), it is unlikely that the results would be consistent across platforms and as such it would be interesting to compare and consider online engagement across multiple platforms relative to broadcast media. Moreover, additional social media platforms (e.g., Facebook Live) and other “unconventional” OTT services (e.g., Amazon Prime) are increasingly moving into the live (sport) broadcasting sphere. As such, future research should continue this investigation by considering other platforms/streaming options and online engagement. Moreover, there could be a comparison between sports, such as the WNBA (streaming on Twitter) and the NFL (streaming on Amazon Prime), as we see new broadcasting deals emerge to understand the intersections between sports, online engagement, and broadcast mediums.

Future scholarship should also consider social media engagement patterns across sport leagues. As a non-traditional professional sport league, the WNBA does not have the same broadcasting rights contracts as more mainstream professional sport leagues in the USA. Consequently, the WNBA might have to leverage unconventional broadcasting channels just to access their fan base and increase their content's reach, resulting in broadcasting rights deals being a necessity to maximize distribution and revenue, rather than a strategic decision based on maximizing engagement. Future research could look to contact Twitter and WNBA team and league executives to better understand the motivating factors behind their partnership. Moreover, future research could also look at the impact of broadcasting medium across different sport leagues, since each league has a unique combination of broadcasting platforms and as such might experience different impacts on online engagement and seek to identify similarities and differences across sport contexts.

Finally, the nature of our social media data only captures a portion of sport consumers' behaviors. It is unclear from our data how or why sport consumers were using social media, how they typically consume WNBA games, or their preferences with respect to the broadcast medium. Future scholarship should consider more in-depth approaches, such as surveys and interviews, to critically examine consumers' opinions and preferences with respect to broadcast options and social media engagement behavior.

## Conclusion

In conclusion, digital streaming of live sport events holds promise for women's sport and niche sports with limited television contracts or resources to produce their own OTT service. Furthermore, advancements in technology and shifting consumer preferences and behaviors have resulted in a number of new broadcast options through which sport leagues can distribute their content directly to fans. This research sought to examine the impact of the broadcast medium on social media engagement with respect to game day WNBA team account posts. Results indicate that athlete/team quality and performance, namely, the presence of an MVP player, the number of all-star players on a team, and being the league champion the previous season, were positively associated with post engagement. However, broadcasting on League Pass or local TV (for home teams) and Twitter were associated with lower post engagement. Since online engagement is associated with offline and online behavioral intentions toward teams and viewership, these findings have important implications for sport leagues seeking to maximize their online engagement and viewership.

## Data Availability Statement

The data analyzed in this study is subject to the following licenses/restrictions: This data set is created from publicly available social media data. Requests to access these datasets should be directed to AP, pegoraro@uoguelph.ca.

## Author Contributions

All authors listed have made a substantial, direct and intellectual contribution to the work, and approved it for publication.

## Conflict of Interest

The authors declare that the research was conducted in the absence of any commercial or financial relationships that could be construed as a potential conflict of interest.
